# Surgery

**Published:** 1843-01

**Authors:** 


### SURGERY.

*On the Treatment of small Nævi in Children, with Acetum Lithargyri.*
By Dr. SIGMUND.

THE author informs us that after the employment of a variety of remedies he finds acetum lithargyri the most powerful in causing to shrink and disappear those vascular growths in children, when the growth does not exceed the size of a hazel nut. This disease attacks the face chiefly; the upper lip more particularly: an operation for its removal is apt to cause great disfigurement; and the method of acupuncture recommended by Lallemand, and compression, seldom prove successful. For small, nonpulsating tumours of this description, the acetum lithargyri, used as a cold fomentation, or applied in small compresses, renewed twice a day, will be found highly useful. It causes a separation of the epidermis, which cracks and falls off; and the newly exposed surface is to be treated as the layer just removed. In four weeks, by this method, the author has succeeded in removing small nævi.

*Oesterreich. Medicin. Wochenschrift.* No. xix, *Mai* 7, 1842.

---

*Analysis of a Calculus removed by Operation from the Lachrymal Canal.*
By M. BOUCHARDAT.

THE calculus occurred in a lady aged sixty-six, who had always been healthy, with the exception of some slight attacks of gout, which had left small concretions on the joints of the toes and fingers.

The calculus weighed 4-10ths of a grain, and yielded on analysis:

| | |
|---|---|
| Concrete albuminous matter | 25 Parts. |
| Mucous matter | 18 |
| Fat | A trace. |
| Carbonate of lime | 48 |
| Phosphate of lime and magnesia | 9 |
| Chloride of sodium | A trace. |

*Annales d'Oculistique. Juillet,* 1842.

### *On Strabismus.* By M. Velpeau.

From this long essay the following statistics alone admit of extract. Of 300 cases on which M. Velpeau has operated, and which have been since watched, he has found that, in half, the remedy was so complete that, except by looking close, it could not have been said that the patient had ever squinted. In the other half, one third was benefited in various degrees, but there was some visible deviation of the eye from its proper axis. In the remaining two thirds of this half, or in one third of the whole, the operation was absolutely unsuccessful; that is, the eye either retained its whole deviation, or had moved too far in the opposite direction, or was turned upwards or downwards, or there was exophthalmia, or immobility of the eye, or some grave defect. Many of these cases M. Velpeau believes owed their ill result to want of attention to his rules after the operation: but the public, he says, had heard so much of the simplicity and certainty of the operation, that it was impossible to persuade them to be cautious.

In 128 of his cases, tabulated by M. Gouraincourt, there were

| | | |
|---|---|---|
| Convergent strabismus in | | 111 |
| Of these 17 were double, | | |
| 67 on the right side, | | |
| 27 on the left. | | |
| Divergent strabismus in | | 17 |
| Of these 7 on the left side, | | |
| 10 on the right. | | |

In another set of 138 cases, there were

| | | |
|---|---|---|
| Convergent | | 123 |
| Double | 20 | |
| Right | 52 | |
| Left | 51 | |
| Divergent | | 15 |
| Double | 2 | |
| Right | 8 | |
| Left | 5 | |

In another set of 45 cases there were

| | | |
|---|---|---|
| Convergent | | 35 |
| Divergent | | 10 |
| Right side | 22 | |
| Left | 23 | |

*Annales de la Chirurgie. Mai,* 1842.

---

### *Statistical Studies on the Results of the Capital Operations in the Hospitals of Paris.* By M. Malgaigne.

This is an excellent paper in the style in which M. Malgaigne is so usually successful, that he would do well to adopt it always in preference to that flippant strain in which he has of late been writing on medical history. After pointing out the improbabilities of the notions commonly entertained respecting the small mortality of amputations performed by certain surgeons (especially Percy and Dupuytren), he proceeds to the statistical analysis of all the amputations performed in Paris from the 1st of January 1836, to the same day in 1841, and of which the total number was 852. We shall follow him in as brief an abstract as will convey the important facts of his analysis, dividing the 852 amputations according to the situations at which they were performed.

*Amputation at the hip-joint,* 1 case, which ended fatally.

*Amputation of the thigh,* 201 cases, of which 126, or nearly 2-3ds, died. Of these 201, 46 were performed for injuries, and 34, or 3-4ths, died; 153 were performed for chronic diseases, and 92, or 3-5ths, died. In one case the amputation was performed on both sides, and the patient recovered. Of the same 153 operated on for diseases, 115 were males, and of these 64 per cent. died; 38 were females, and 53 per cent. died; 24 were between 5 and 15 years old, and 8 of them died; 90 were between 15 and 35 years old, and 52 of them died; 39 were between 35 and 70 years old, and 32 of them died. On the other hand, of the 46 operated on for injuries, 4 were between 5 and 15 years old, and all died; 26 were between 15 and 35, and 18 of them died; and 16 were between 35 and 70, and of these 12 died.

*Amputation of the knee-joint.* Of 9 patients on whom this operation was performed, 7 died. Of 14 cases formerly recorded, M. Velpeau had found only 1 fatal!

*Amputations of the leg.* The total number was 192, of which 106, or about 55 per cent. were fatal. For diseases, 113 were performed, and 55, or half, were fatal; for injuries 79, of which 50, or nearly 2-3ds, were fatal. The patients were 80 males, of whom 46 per cent. died; and 32 females, of whom 56 per cent. died, when the amputation was performed for diseases; while of those who had received injuries there were 67 men, of whom 62 per cent. died; and 12 women, of whom 66 per cent. died. Of the former class also, there were 21 between 2 and 15 years old, of whom 5 died; 48 between 15 and 35 years old, of whom 25 died; and 43 between 35 and 76, of whom 25 died; while of the latter class (who had suffered injuries), 4 were between 2 and 15, and 3 died; 35 were between 15 and 35, and 19 died; and 40 were between 35 and 76 years old, of whom 28 died.

*Partial amputations of the foot.* Of 38 cases, 9 were fatal, or 24 per cent.; of these, 29 were performed for chronic diseases, and only 3 were fatal; while of the other 9 performed for injuries, 6 were fatal. Of the 29 patients, 20 were under 35 years old, and all recovered; 9 were upwards of 35, and 3 died. Of the 9 who had received injuries, 4 were between 27 and 37 years old, of whom 1 died; and 5 were between 35 and 58 years old, and all these died.

*Amputation at the shoulder-joint.* There were 14 of these performed in the five years. One patient had his thigh amputated at the same time and died. Of the remaining 13, 10 died; 7 of these had received injuries, and all died.

*Amputation of the upper arm* was performed 91 times, and there were 41 deaths, or about 45 per cent. In 61 of the patients there had been previous disease, and of these, 24 or 2-5ths died; the 30 others had received injuries, and of these 17, or more than half, died. Of the 61, 9 were between 4 and 15 years old, and 3 died; 34 were between 15 and 35 years old, and 13 died; 18 between 35 and 80 years old, and 8 died; while of the 30 injured patients, 1 was between 5 and 15, and died; 10 were between 15 and 35, and 7 died; and 19 were between 35 and 84 years old, and 9 died.

*Amputation of the forearm.* 28 were performed, and 8, or 28 per cent., were fatal. Of the whole number of patients, 17 had had disease below the amputation, and 5 died; 11 had received injuries, and 3 died. As to age, 3 of the diseased were between 5 and 15, and 1 died; 4 were between 15 and 35, and 1 died; 10 were between 35 and 70, and 3 died; while of the injured, 1 was 8 years old and recovered; 3 were between 15 and 35 and all recovered; 7 were between 35 and 70, and 3 died.

*Amputation at the wrist-joint* was performed 16 times, and all the patients recovered; 4 of the patients were women and 12 were men; 4 had been injured and 12 diseased; their ages varied from 11 to 65. Of all the great amputations, therefore, this was least fatal.

*Amputation through one or more of the metatarsal bones* was performed 8 times (exclusive of venesection and extirpations), and 1 patient, a man who had received an injury of the toe, died. *A metacarpal bone* was amputated 9 times, and 1 patient, a man æt. 22, who had had disease of the part removed, died. *Amputation of the great toe* by disarticulation was performed 43 times, and 7 of the patients, or 1-6th, died. Of the whole number, 29 had disease of the toe, and only 3 died; while of the 14 who had received injuries of it, 4 died. All those who died were men; 5 women recovered. Of the *pathological* amputations, 2 were performed on patients between 5 and 15 years old, and both recovered; 18 were between 15 and 35, and 1 died; 9 were between 35 and 70, and 2 died; while of the *traumatic* cases, 6 patients were between 15 and 35, of whom 1 died; and 8 were between 35 and 70, of whom 3 died. *Amputation of one of the smaller toes* was performed 26 times, 12 of the operations being for injuries, and 1 patient, a man 40 years old, with disease of the toe, died. *Amputation of several toes* at once was performed 7 times, and there was 1 death. *Amputation of the phalanges* was in 9 cases successful, in none fatal.

*Amputation of the thumb* was fatal in 3 cases out of 9, all the 3 operations having been performed for injuries. *Fingers* were amputated singly 119 times, and in 10 cases death followed; namely, in 6 out of 79 pathological cases; and in 4 out of 40 traumatic cases. *Several fingers* at once were amputated 13 times, and death ensued in 1 case. *Phalanges* were amputated 24 times, and death ensued once.

The general results of these statistics may now be briefly summed up. Amputations after injuries are more frequently fatal than those performed for chronic diseases. The mortality of the former is 64 per cent. in the greater, and 15 per cent. in the minor amputations; that of the latter is 48 per cent. in the greater, and 7½ per cent. in the minor.

After injuries, M. Malgaigne has found that of 49 immediate amputations, 34 were fatal; and of 20 secondary amputations, 13.

Females recover from amputations more often than males. The proportionate mortality after great amputations is for males 55 per cent., for females 47 per cent.; and after minor amputations, for males 12¼, for females 4 per cent. About the same ratio holds whether the amputation be performed for injury or for disease.

For the influence of age, nearly the most unfavorable period is that of infancy; from 5 to 15 years of age the mortality is less, and this is, on the whole, the most favorable period for both sexes; beyond this age the mortality gradually increases, and more rapidly in women than in men; from 50 to 65 is the most unfavorable period for both sexes; but after 65 the mortality seems to decrease.

In the different seasons, the mortality increases in the following order, winter, summer, spring, autumn: in the first it was (in the cases analysed, which were all great amputations), less than 50 per cent.; in the last about 55 per cent. Slight variations are found according to the classes of cases analysed, but the same general order is maintained, except in the case of patients from 15 to 20 years old, to whom the winter is the most destructive season, and then autumn, summer, and spring successively.

An analysis is lastly attempted in order to determine the comparative ratio of mortality in each of the chief Parisian hospitals; but its results are not definite.

*Archives Génerales de Médecine. Avril et Mai,* 1842.

---

*On the Fractures of the Lower End of the Radius.* By M. Voillemier.

The chief object of this very excellent paper is to prove, especially by examination after death, the direction which fractures of this kind usually take, and thence to deduce the best method of treating them. The difficulty and importance of the question is sufficiently proved by the number of essays which have been recently published upon it. We can only abstract the peculiar portions of the author's views.

As the basis of these he points out some facts in the structure of the radius, namely, the double inclination of its inferior articular surface, which is oblique from above downwards and from before backwards, and from within outwards; its prominent border behind and on the outer side; and, above all, the thinness of the compact tissue of the lower extremity of the radius, when compared with that of the shaft, the former becoming suddenly almost as thin as paper, while the latter is so thick that there is scarcely any medullary tube. It is from this last circumstance that there results both the peculiar tendency to fracture near the wrist, and the position in which, according to M. Voillemier, the fragments are usually placed, and which is such that the fracture may be called *fracture by penetration*.

In a fall on the hand (the most frequent cause of this fracture), all the force of the shock is transmitted to the radius, and it breaks at its weakest point, where the compact tissue is thinnest, and *that* is just above the wrist-joint. At this part, too, the cancellous tissue is first abundant; the compact tissue of the wall of the upper portion is therefore, by the continuance of the force after the fracture has taken place, driven into the cancellous tissue of the lower. This penetration may take place in several ways. If the extremity of the bone is large, and the force has been transmitted in nearly the direction of its axis, and all parts of the wall have yielded almost simultaneously, the whole of the end of the superior fragment may be driven into the lower one, and the two pieces may be thus locked or *nailed* one within the other. When, in such a case, the force is very great, the upper fragment may be so driven into the lower, that the latter may be completely smashed; it was to cases like this that Dupuytren gave the name of *fractures par ecrasement.*

But this mode of fracture, as might be expected from the number of circumstances which must combine to produce it, is rare; the more common form is produced when the force of the fall comes somewhat obliquely upon the radius. In this case, the line of fracture is, as in the last, nearly parallel to the plane of the articular surface, but since the force falls more on the posterior than on the anterior part of the latter, it follows that the posterior edge of the fractured compact tissue of the upper fragment begins first to penetrate into the cancellous tissue of the lower, and that the anterior edge, instead of penetrating, slides over upon the dorsal surface of the lower or carpal fragment. There is thus a dovetailing or reciprocal penetration of the two portions. And a similar arrangement of parts is seen, whether one traces them from behind forwards, or from without inwards. In the former direction, one comes first on the dorsal portion of the upper fragment, then on the same portion of the lower fragment, and then, in the same succession, on their palmar portions; and in the latter direction there is, on the outside the outer margin of the lower portion, then the outer margin of the upper one, and so on; for the outer edge of the upper portion in these cases always penetrates the cancellous tissue of the lower in such a manner that, if the line of its wall were carried on, it would cut off the styloid process. Hence it is that in all the cases of fracture of the radius near the wrist which M. Voillemier has examined long after the accident, he has found on a vertical section of the bone, a line of compact tissue continued straight downwards from the outer wall of the shaft into the interior of the cancellous tissue of the head. There are not two such lines of compact tissue, because that of the lower portion which should penetrate into the upper, is usually broken up and absorbed, in consequence of its extreme thinness.

Having found this state of parts in the great majority of instances, M. Voillemier denies the existence of oblique fractures of the lower end of the radius, and of those especially which M. Diday had described as fractures "taillés en biseau." These, he says, have been supposed to exist in consequence of the sensation which fragments, placed as described above, give when examined during life. The dorsal edge of the upper fragment projects somewhat lower down than the palmar edge of the lower one, for the former is pushed downwards over the lower fragment, the latter upwards over the upper one; and hence it seems to the touch as if the line of fracture were oblique from the palmar to the dorsal surface, and from above downwards. But such a state of parts has never been found on dissection. The condition is analogous in those rarer cases in which the lower fragment passes, not towards the dorsal but towards the palmar aspect of the upper one. Here there is only a seemingly oblique direction of the fracture, in consequence of the projecting edge of one fragment being higher up than that of the other.

From these pathological facts, which we believe to be exactly conformed to the truth, M. Voillemier gives a clear *rationale* of the symptoms, and then deduces the mode of treatment best to be followed. The chief feature of that which he recommends is its simplicity, but he, without doubt, asserts a true principle when he says that in these fractures, even more than in most others, the apparatus must be adapted differently to almost every case.

An important addition to this paper is that part in which M. Voillemièr speaks of the separation of the inferior epiphysis of the radius which may take place in consequence of severely twisting the wrist-joint. He was led to notice this, by having accidentally produced it in an attempt to dislocate the wrist of a dead body; and he subsequently convinced himself that either this separation, or a fracture of the lower end of the radius, without complete rupture of the surrounding fibrous tissues, might be thus produced during life. He relates two cases in which he believes it had occurred, and points out, as its chief signs, the absence of deformity where all the other characters of fracture are present, and a most severe pain at the ordinary situation of the boundary between the epiphysis and shaft, whenever any attempt at motion is made.

*Archives Générales de Médecin. Mars,* 1842.

---

*On Extirpation of the Eye.* By Dr. Bonnet, of Lyons.

The attention lately paid to the orbital capsule of Tenon, has led Dr. Bonnet to an improved method of extirpating the eye, in those cases in which nothing but the eyeball requires to be removed. In the ordinary mode of operating, the instrument is buried in the fat of the orbit, and the muscles are divided at a considerable distance from their insertion into the eyeball; the trunks of the nerves are cut across, as well as considerable branches of the ophthalmic artery. All this is avoided, if the muscles and the optic nerve are divided at their insertion into the sclerotica, and the eyeball removed, leaving Tenon's capsule entire. In this mode of operating, all risk of hemorrhage is avoided, the optic nerve only is divided, and the wound is separated from the remaining contents of the orbit by the capsule. Separating the eyelids, Dr. Bonnet proposes to divide the rectus internus, as in the operation for strabismus; next, sliding the scissors through the wound, they are to pass between the sclerotica on the one side, and the subconjunctival fascia and muscles on the other, and to divide the remaining three recti close to their insertions; the two obliqui are to be divided as near as possible to the eye; then, the optic nerve. The eye is now removed, without implicating any vessel, or any other nerve than the optic, and without penetrating into the fat of the orbit.

The cases suitable for this mode of operating must be few, the textures surrounding the eyeball being in general too much implicated in the disease to be left behind. Gensoul had a case, in which Bonnet's plan might have sufficed. The eye was changed neither in form nor size; vision was destroyed; but the excruciating pain suffered by the patient, and which no means were found to mitigate, was the only circumstance which determined the question as to an operation. A melanotic tumour, confined to the retina, was found to be the disease.

Dr. Stœber, of Strasburg, has removed an eye affected with melanosis, nearly on Bonnet's plan. Having cut the rectus internus, he drew the eye forwards, divided the optic nerve with curved scissors, and finished the operation by separating from the eye the conjunctiva and the muscles. The eye, thus removed, looked as if nicely dissected, the sclerotica being perfectly free of cellular substance. The operation required much less time than the ordinary extirpation, and very little blood was lost. Dr. Cunier has removed an eye according to the same method. The eyeball was not changed in form; the disease was medullary fungus. The suppuration was very great, and the granulations excessive. Experience must determine, whether these effects are generally to be expected when this mode of operating is adopted.

*Annales d'Oculistique. Avril,* 1842.

*Removal of a whole Rib.* By Signor METAXA, of Rome.

THIS operation was performed for necrosis, which had long existed, and had originated in a wound, with violence inflicted on the tenth rib. There had been reducing suppuration, and the author had proposed to remove a portion only of the rib, but he found that the necrosis extended so much further than he expected, that he was obliged to disarticulate the head of the rib from its connexion with the vertebra. The patient quickly and completely recovered.

*Annali Medico-Chirurgici. Aprile,* 1842.

---

*Vaccination and Revaccination.* By Dr. AXISA, of Malta.

GREAT difficulty having been experienced in maintaining a supply of good vaccine lymph in Malta, Dr. Axisa was commissioned to make experiments to discover if vaccine lymph inoculated in the cow was reproduced; and if so, whether it maintained its salutary properties. In this inquiry the Doctor was assisted by a medical committee. He performed his first experiment 15th June, 1841. With lymph from the pustules of a healthy girl two years of age, who had been vaccinated seven days, he inoculated the udder of a cow in thirteen points. On the 9th there was a distinct areola around each spot; 11th, pustules full of lymph, and circumscribed by an areola, livid red, tense, and swollen. On the 13th the pustules were examined by a number of physicians, who all agreed that they were the true vacciniæ, the areola however being wanting, which Dr. Axisa says disappeared that day.

A pustule was opened. It contained liquid lymph, with which ten children were vaccinated on the same day. 16th, the pustules on the cow had desiccated. Of the ten children inoculated only two had the vaccine *eruption, all had a single pustule,* and in one the pustule was well developed. On the 8th day, and with the pus from this pustule another cow was vaccinated in sixteen points, and on the same day with the same matter a child three months old. On June 22 the pustules of the first two boys were flat, umbilicated, and surrounded by their respective areolæ. On the 28th, the pustules of the second cow, and of the child inoculated on the 21st, were undeveloped and areolated. Five children were inoculated with the matter from these pustules, and one of them with this matter in one arm, and in the other arm with that of the pustules of the boy inoculated at the same time with the second cow. On July 6th, the pustules in all were well developed, and in one there was the vaccine eruption.

*Il Filocamo. Febbrajo* 15, 1842.

---

*New Mode of Extraction of Cataract.* By Dr. BONNET, of Lyons.

HE lays the patient on his back. If it is the left eye, he places himself at the patient's right side, and having applied the elevator and depressor to the eyelids, intrusts them to two assistants, who must see that they do not press upon the eyeball. With his left hand, the operator holds a common pair of forceps; with his right, a hooked spring pair. With the former, he raises the conjunctiva into a fold, and with the latter takes firm hold of that membrane, and of the subconjunctival fascia about one sixth of an inch from the cornea, a little above the external angle of the eyelids. This forceps is then intrusted to the assistant who supports the upper eyelid; it fixes the eye and prevents it from rolling inwards. This operator now takes the depressor of the lower lid into his left hand, and with the right uses the knife to divide the cornea.

To the mode of fixing the eye, above explained, he ascribes the promptitude, precision, and facility, with which the section is accomplished. The incision is always parallel to the edge of the cornea; the iris is never wounded; the operation is as simple, he says, as bleeding: no dangerous consequences ever arise from the pinching of the conjunctiva with the forceps; the specula applied to the eyelids prevent pressure on the eyeball, and that dangerous expulsion of vitreous humour which is apt to happen when the fingers are employed to fix the eye and separate the eyelids! All this we owe to the operation for strabismus!

Dr. Pétrequin employs Bonnet's method of fixing the eye, in the operations of displacement, and in those for artificial pupil.

*Annales d'Oculistique. Avril,* 1842.

*Cure of Asthenic Amaurosis by the use of Convex Spectacles.*

Taking a hint from certain quack vendors of spectacles, Dr. Cunier of Brussels has made trial of the effects of convex glasses, in what he terms *cases of simple anæsthesia of the retina,* and with a considerable share of success. The influence of the light, directed upon the retina by such glasses, appears beneficially to excite the sunken sensibility of the optic nerve.

A lady, whose case he relates, could not for eight years without difficulty distinguish with her left eye the large characters forming the title of a newspaper; could not tell the hour on the clock, unless her eye was within two inches of the hands, nor distinguish the feature nor the figure of a person at the distance of two feet. If the right eye was covered, the left pupil became dilated, and remained so.

Glasses, whether concave or convex, ought always to be distinguished by their focal lengths, and not by numbers. As Dr. Cunier employs the latter mode, we are at a loss to know the precise power of No. 3, with which the patient could read a large type with her left eye, but probably it was a convex lens of three inches focus. After exercising the eye with this lens for some minutes, vision became confused and the head painful, so that the patient was obliged to desist. Next day she could read with No. 3½, and on the third day with No. 4. The duration of this kind of exercise was gradually lengthened, and glasses of longer and longer focus employed. By the tenth day, the patient read the hour on the clock at the distance of 22 inches, and recognized individuals at double that distance. By the seventeenth day of the treatment, No. 24 was employed, and the patient could read small type. After two months' use of No. 24, the sight of the left eye was as good as that of the right.

Several other successful cases are related by Dr. Cunier.

*Annales d'Oculistique. Mai,* 1842.

---

*Cases of Hepatic Abscess Explored and Punctured.*

In our 23d Number, (p. 89 et seq.), we have referred to exploratory puncture of the liver, and expressed our opinion as to the manner and circumstances in which the operation is to be performed. We find that this method of treating hepatic abscess appears to gain ground with our medical brethren in the east; and that, to use the words of the editors of the Madras Quarterly Medical Journal, "it offers almost the only chance which remains for the patient suffering under liver abscess." (vol. iii. p. 394.) The operation itself, when skilfully performed, appearing to be devoid of danger, and to lead to no bad consequences, nor to aggravate, in any manner, the circumstances of the patient, is not only as justifiable as any other surgical operation, but seems to be imperatively indicated, in all cases in which abscess is suspected, and its site can be guessed at.

Private Squires, aged twenty-five, was admitted to hospital on the 6th of April, 1840, with acute pain in the right hypochondrium, increased by either pressure or full inspiration. He was treated with purgatives, mercurials, and blisters, but without success; for on the 28th, a tumour presented itself posteriorly in the lumbar region. On the 1st of June, the exploratory needle was passed into the tumour, and on pus flowing, a trocar was introduced, which gave vent to ten ounces of healthy matter, to the great relief of the patient. The canula was retained, and in the course of the afternoon, ten ounces more were discharged. The wound continued to discharge, though slightly, until the 26th, and on the 21st of August, the man was discharged to duty, cured.

In the next case, the explorator was passed twice, without matter being detected, but without any bad consequences resulting from the operation. But the man died, and on examination, two large distinct abscesses were detected in the right lobe of the liver, and three small ones in the left.

The operation, as we have already said, seems to be a perfectly safe one, effusion into the abdomen never taking place in consequence of it. The following is an account of the appearances, on post-mortem inspection, of a case in which deputy-inspector Murray successfully explored and punctured an hepatic abscess: the man recovering from the liver affection, and dying, two years subsequently, from dysentery.

"On dividing the muscles of the belly, the liver appeared larger than usual, and of rather a darker colour: there were no adhesions to any part of the abdominal parietes, except at the point where the puncture had been made; and at this spot it appeared as if it was attached to the walls of the abdomen, or rather hung from it by a cord, about the size of a small goose quill. On dividing its substance minutely, not the slightest vestige of the sac of an abscess could be found, or anything that would indicate such having existed."

*Madras Quarterly Medical Journal.* Vol. iii.

